# Sir Dyce Duckworth on Diatheses

**Published:** 1901-11-16

**Authors:** 


### SIR DYCE DUCKWORTH ON DIATHESES.

Sir Dyce Duckworth would revive the old term scrofula, applying it to those who are specially prone to be attacked by tuberculosis. It is now deliberately asserted, he says, by many pathologists that there is no such condition as is denoted by the words scrofula and struma, that in fact struma is neither less nor more than tuberculosis. From all such teaching, however, he dissents. It cannot be denied, he says, that the liability to tuberculous disease is limited; that certain persons are distinctly more prone to fall a prey to it than others, and that happily the majority of mankind present a resistance to it, no doubt varying in degree, but in many cases amounting to practical immunity. We have then to discover the peculiarities of these persons whose bodies form a suitable nidus for the development of tubercle, and on the other hand of those who present so marked a resistance to the infection. The first are the scrofulous or strumous. Sir Dyce Duckworth maintains that there is a class of persons who by inheritance, or by acquirement owing to various debilitating conditions, are frail and delicate, endowed with a specific proclivity to become gravely affected by irritants of all kinds, and especially prone to infection by tubercle bacilli. These persons are of delicate constitution and are apt to manifest this delicacy in various ways throughout life, such developments indicating their scrofulous habit of body. They may never become tuberculous although always scrofulous. The latter proclivity may blend with other habits of body and seriously modify the ailments induced by them. We have not to wait for tuberculous invasion before we pronounce them scrofulous, since they carry with them the features and the characteristics of the scrofulous condition beforehand. The ailments of the scrofulous individual are not necessarily of a tuberculous quality, and the error in modern teaching is, as Sir Dyce Duckworth believes, in asserting that this is the case. Coming now to those who display a marked resistance to tuberculosis he instances those who manifest a proclivity to gout. In the offspring of gouty parentage and ancestry it is most rare to meet with any strumous indications or disorders. Tuberculosis in a gouty subject is rarely witnessed, and its progress is either very slow or apt to be arrested. "There is thus a manifest antagonism between a gouty and a strumous proclivity. We may further note that a line of treatment which is appropriate to those who are goutily disposed is unsuitable for strumous subjects, and that what is bad for the gouty is good for the scrofulous—to wit, animal food and wine. The presence of a strain of gout in a strumous subject is thus a saving grace for the individual, modifying the tendency and course of any manifestation of the latter in a favourable way."

